# Clinical value of ultrasound combined with the nutritional risk index in predicting the efficacy of neoadjuvant chemotherapy for breast cancer: An observational study

**DOI:** 10.1097/MD.0000000000047070

**Published:** 2026-01-23

**Authors:** Yan Shen Liu, Zuo Jie Li, Hui Wang, Jiao Qun Zhou

**Affiliations:** a Department of Ultrasound, The First People’s Hospital of Fuyang Hangzhou, Hangzhou, Zhejiang Province, People’s Republic of China; b Department of Clinical Laboratory, The People’s Hospital of Cangnan Zhejiang, Wenzhou, Zhejiang Province, People’s Republic of China; c Department of Pathology, The First People’s Hospital of Fuyang Hangzhou, Hangzhou, Zhejiang Province, People’s Republic of China; d Department of Breast Surgery, The First People’s Hospital of Fuyang Hangzhou, Hangzhou, Zhejiang Province, People’s Republic of China

**Keywords:** breast cancer, neoadjuvant chemotherapy (NAC), nutritional risk index (NRI), pathologic complete response (pCR), ultrasonography

## Abstract

The early prediction of neoadjuvant chemotherapy (NAC) efficacy is essential for the timely modification of treatment regimens. This study investigates the clinical utility of integrating ultrasound examination with the nutritional risk index (NRI) to predict pathological complete response (pCR) in breast cancer patients following NAC. Utilizing a retrospective analysis, we examined data from 85 breast cancer patients who underwent NAC between January 2020 and December 2023. All participants received routine ultrasound evaluations and NRI assessments both prior to NAC initiation and after the completion of 2 treatment cycles. Based on the Miller–Payne grading system, patients were categorized into the pCR group (39 cases) and the non-pCR group (46 cases). Significant variables (*P* < .05) were identified through univariate logistic regression analysis and subsequently incorporated into a multivariate binary logistic regression model (inclusion criterion: *P* < .20). The diagnostic performance was assessed using the receiver operating characteristic curve, while the calibration curve was utilized to further evaluate the predictive value. The multivariate analysis identified 3 independent predictors of pathological complete response (pCR): N stage (N2: odds ratio [OR] = 3.68, 95% confidence interval [CI]: 1.08–12.53, *P* = .037), change in tumor maximum diameter after the second neoadjuvant chemotherapy (NAC) cycle (ΔD2; OR = 0.95, 95% CI: 0.92–0.99, *P* = .024), and change in nutritional risk index after the second NAC cycle (ΔNRI2; OR = 1.10, 95% CI: 1.01–1.19, *P* = .032). The variance inflation factor values (ranging from 1.070 to 1.163) indicated an absence of multicollinearity. The combined diagnostic model achieved an area under the receiver operating characteristic curve of 0.847 (95% CI: 0.765–0.930), with a sensitivity of 69.2% and a specificity of 89.1%. The calibration curve demonstrated strong agreement between predicted and actual outcomes (Hosmer–Lemeshow test, *P* = .747). The integration of N stage, ΔD2, and ΔNRI2 exhibits significant clinical utility for the early prediction of NAC efficacy in breast cancer, potentially informing treatment adjustments and enhancing patient outcomes. However, the study’s small sample size (n = 85) and single-center retrospective design constrain its generalizability. Therefore, large-scale, multicenter prospective validation is necessary prior to clinical implementation.

## 1. Introduction

Breast cancer represents the most common malignant tumor among women worldwide and constitutes a major public health concern.^[1,2]^ GLOBOCAN 2022 reports that breast cancer is the second most diagnosed cancer globally, with 2.3 million new cases and 6,66,000 deaths.^[3]^ It is the most common cancer and leading cause of cancer death among women worldwide. The global incidence rate is 46.8 per 1,00,000 women, varying significantly by region.^[3,4]^ In China, breast cancer is the top cancer among women, with 3,57,200 new cases and 75,000 deaths in 2022.^[4,5]^ The incidence rate in China is 33.04 per 1,00,000, lower than in Japan and other developed East Asian countries.^[4]^ The increasing incidence highlights the need for better treatment strategies and prediction methods.^[3]^

Various treatment modalities are available for breast cancer, including surgical intervention, radiotherapy, chemotherapy, and targeted therapy.^[6,7]^ Neoadjuvant chemotherapy (NAC) is a critical therapeutic strategy, particularly valuable for patients with larger tumors or those at high risk of recurrence.^[8]^ Research indicates that neoadjuvant chemotherapy can effectively reduce tumor size, enhance the feasibility of breast-conserving surgery, and improve long-term survival outcomes.^[9,10]^ In patients with triple-negative breast cancer (TNBC), neoadjuvant chemotherapy has demonstrated notably significant effects. Comparative studies on the prognostic benefits of neoadjuvant chemotherapy versus adjuvant chemotherapy in TNBC patients reveal that neoadjuvant chemotherapy substantially improves disease-free survival in individuals with stage IIb to IIIa TNBC. These findings underscore the pivotal role of neoadjuvant chemotherapy in specific breast cancer subtypes.^[11]^ Furthermore, neoadjuvant chemotherapy has demonstrated a beneficial effect on the surgical outcomes and prognosis of breast cancer patients.^[10]^ In summary, neoadjuvant chemotherapy is a crucial component in the management of breast cancer, particularly for patients with large tumors and a high risk of recurrence, offering significant clinical advantages in terms of tumor reduction, increased rates of breast-conserving surgery, and improved long-term survival.

Neoadjuvant chemotherapy (NAC) is frequently employed in the treatment of breast cancer; however, patient responses to this therapy vary considerably. Current evidence indicates that the effectiveness of NAC ranges from 60% to 85%, with approximately 5% of patients experiencing disease progression during treatment.^[12]^ At present, pathological assessment is regarded as the gold standard for evaluating the efficacy of NAC. Nonetheless, the results of such assessments often lag behind the treatment process, complicating the timely adjustment of therapeutic strategies. Consequently, the early and accurate evaluation of NAC efficacy is of paramount importance.^[13]^ Recently, researchers have extensively investigated early prediction methods for assessing NAC efficacy. For instance, prediction models that integrate ultrasound imaging with clinicopathological features are utilized to evaluate pathological complete response (pCR) in breast cancer patients undergoing NAC, demonstrating high predictive accuracy.^[12]^

In addition to the patient’s condition, nutritional status is also a crucial factor in determining the effectiveness of breast cancer treatment. Cachexia is a common metabolic syndrome characterized by muscle wasting, fat redistribution, and metabolic disorders, typically associated with advanced cancer but sometimes seen in early stages as well. This syndrome significantly impacts patient outcomes and is linked to poor chemotherapy responses and reduced survival rates.^[14]^ Cancer-related malnutrition leads to poorer chemotherapy tolerance, higher toxicity, and weakened immune function, resulting in less effective treatment.^[15,16]^ Malnourished patients face more treatment interruptions and complications due to reduced physiological reserves, altered drug metabolism, and inflammation.^[16–18]^ Early nutritional assessment and interventions are crucial for enhancing treatment tolerance and outcomes.^[15,16]^

In the nutritional assessment of cancer patients, although BMI and serum albumin are commonly used indicators, they have certain limitations in comprehensively reflecting the patient’s nutritional status. The nutritional risk index (NRI) serves as a comprehensive instrument that evaluates a patient’s ideal weight, current weight, and fluctuations in serum albumin levels, thereby facilitating a nuanced assessment of nutritional status and its prognostic significance concerning nutrition-related morbidity and mortality among cancer patients.^[19]^ Studies have indicated that a low preoperative NRI is associated with an unfavorable prognosis and an increased risk of postoperative complications, underscoring its potential utility as a prognostic marker for elderly patients with colorectal cancer.^[20]^ Moreover, the NRI has been identified as a predictor of survival outcomes in breast cancer patients undergoing neoadjuvant chemotherapy.^[21]^ Nevertheless, there is a notable paucity of research examining its effectiveness in breast cancer patients receiving neoadjuvant chemotherapy.

This study presents novel opportunities for the early prediction of neoadjuvant chemotherapy (NAC) efficacy in breast cancer patients through the integration of clinical indicators, ultrasound imaging features, and nutritional risk index (NRI). By leveraging the strengths of these methodologies, the study facilitates the earlier identification of patients who are nonresponsive to chemotherapy, thereby providing a foundation for timely adjustments to treatment regimens. This approach aims to circumvent ineffective therapies and optimize personalized treatment strategies, ultimately enhancing survival outcomes and quality of life.

## 2. Materials and methods

### 2.1. Subjects and methods

A retrospective study was conducted to analyze data from a cohort of 85 female patients diagnosed with breast cancer, all of whom received neoadjuvant chemotherapy (NAC) between January 2020 and December 2023. The inclusion criteria for this study were as follows: patients aged between 18 and 75 years; a pathological confirmation of breast cancer; no prior tumor-related treatments before the initiation of NAC, with completion of the planned NAC cycles; availability of clear pathological results and comprehensive immunohistochemical data prior to NAC; and performance of breast ultrasound and routine blood tests assessing albumin and globulin levels within 1 week before the commencement of NAC and after 2 cycles of NAC. The exclusion criteria included: any prior treatment for breast cancer; the presence of other malignant tumors; bilateral or recurrent breast cancer; incomplete ultrasound or circulating tumor cell detection data; and severe dysfunction of the heart, liver, or kidneys, or the presence of other serious comorbidities. This study was conducted with approval from the Ethics Committee of The First People’s Hospital of Fuyang Hangzhou (No. 2025-LW-025). This study was conducted in accordance with the declaration of Helsinki. Written informed consent was obtained from all participants.

### 2.2. Sample size estimation

The sample size for this retrospective study was determined by the availability of eligible patients who met the inclusion and exclusion criteria within the study period (January 2020–December 2023). For the development of a prediction model using logistic regression, the adequacy of the sample size was assessed using the events per variable (EPV) criterion. Drawing on previous studies on the prediction of neoadjuvant chemotherapy response in breast cancer,^[12,21]^ and taking into account the anticipated pathologic complete response (pCR) rate observed in our pilot data, a target EPV of at least 10 was established with 3 to 4 candidate predictors. This required a minimum sample size of approximately 75 to 80 patients. Ultimately, 85 patients who met all eligibility criteria were included in the final analysis, resulting in an EPV range of 8.5 to 11.3, which is considered sufficient for the development of a stable logistic regression prediction model.

### 2.3. Interventions

**Figure 1.**
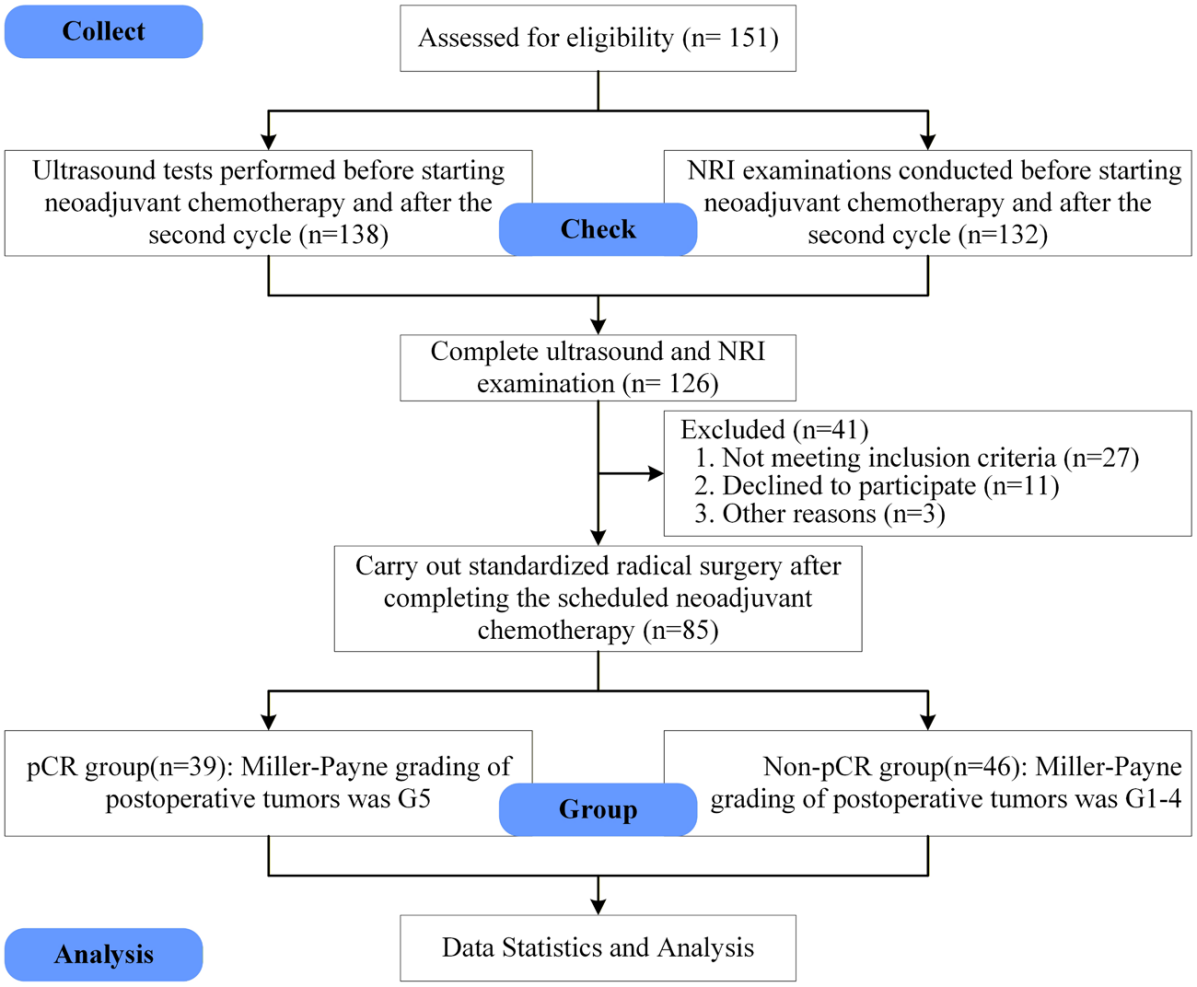
Design and flow of participants through the study. NRI = nutritional risk index, pCR = pathological complete response.

All patients underwent neoadjuvant chemotherapy (NAC) followed by surgical intervention. The NAC regimens were tailored according to molecular subtypes, adhering to the Guidelines for Breast Cancer Diagnosis and Treatment by the China Anti-Cancer Association (2024 edition).^[22]^ Specifically, for HER2-positive disease, patients received chemotherapy regimens based on anthracyclines and/or taxanes, in conjunction with anti-HER2 targeted therapies, namely trastuzumab and pertuzumab, to achieve dual HER2 blockade. For those with triple-negative breast cancer (TNBC), anthracycline- and taxane-based chemotherapy was administered, with the addition of carboplatin and/or pembrolizumab for eligible high-risk patients, based on individualized assessments. In cases of hormone receptor-positive/HER2-negative disease, patients were treated with anthracycline- and taxane-based chemotherapy (such as the AC-T regimen) or the docetaxel and cyclophosphamide regimen. The determination of treatment regimens was conducted by a multidisciplinary tumor board, comprising medical oncologists, surgeons, radiologists, and pathologists. This decision-making process considered tumor characteristics – including clinical stage, molecular subtype, and histologic grade – alongside patient performance status, comorbidities, and individual preferences. Treatment response was systematically monitored. Following chemotherapy, all patients underwent surgical intervention, and their pathological responses were assessed using the Miller–Payne grading system. Grades G1 to G4 were indicative of a non-pathological complete response (non-pCR), whereas grade G5 denoted a pathological complete response (pCR), as depicted in Figure 1.

### 2.4. Routine ultrasound examination

**Figure 2.**
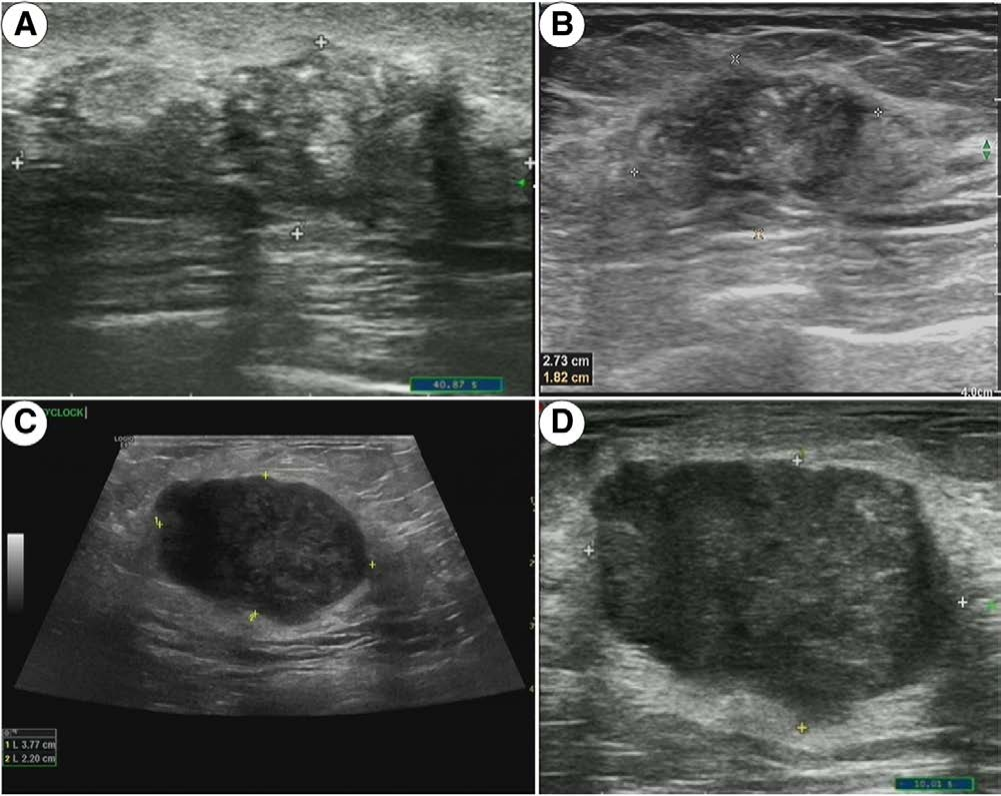
Ultrasound change of breast cancer before and after treatment of neoadjuvant chemotherapy. (A) Ultrasound image of breast cancer before neoadjuvant chemotherapy (NAC) in patients with pathological complete response (pCR), lesion size 43 mm × 15 mm; (B) ultrasound image of breast cancer after the second cycle of NAC treatment in patients with pathological complete response (pCR), lesion size 27 mm × 18 mm; (C) ultrasound image of breast cancer before neoadjuvant chemotherapy (NAC) in patients with non-pathological complete response (pCR), lesion size 38 mm × 22 mm; (D) ultrasound image of breast cancer after the second cycle of NAC treatment in patients with non-pathological complete response (pCR), lesion size 37 mm × 42 mm. NAC = neoadjuvant chemotherapy, pCR = pathological complete response.

Breast ultrasound examinations were performed 1 week prior to neoadjuvant chemotherapy (NAC) and within 1 week following the second chemotherapy cycle. Lesion measurements were conducted in accordance with the 6th edition of the 2023 American College of Radiology’s Ultrasound Breast Imaging Reporting and Data System (BI-RADS-US).^[23]^ In cases of multifocal masses, the largest lesion was measured. The long diameter (D) of the breast cancer lesion was documented, and the percent change in long diameter (ΔD2) was calculated using the following formula: ΔD2 = [(D before NAC) − (D after the second NAC cycle)]/(D before NAC) × 100%. In 1 instance of breast cancer, ultrasound imaging revealed a tumor measuring 43 mm × 15 mm prior to the initiation of neoadjuvant chemotherapy (Fig. 2A). Subsequent to 2 cycles of neoadjuvant chemotherapy, the tumor dimensions decreased to 27 mm × 18 mm (Fig. 2B), and pathological analysis confirmed a complete pathological response. In a separate case, a breast cancer tumor was measured at 38 mm × 22 mm on ultrasound before neoadjuvant chemotherapy (Fig. 2C). After 2 cycles of treatment, the tumor size was reduced to 37 mm × 24 mm (Fig. 2D); however, pathological evaluation indicated an incomplete pathological response. Tumor (T) and nodal (N) staging were determined based on the TNM criteria as outlined in the breast cancer diagnosis and treatment guidelines.^[24]^ Specifically, T1 corresponds to a maximum tumor diameter of 20 mm or less, T2 pertains to diameters >20 mm but ≤50 mm, and T3 applies to diameters exceeding 50 mm. Axillary lymph node staging is defined as follows: N1 includes 1 to 3 lymph nodes with metastases, N2 encompasses 4 to 9 lymph nodes with metastases, and N3 comprises 10 or more lymph nodes with metastases.

### 2.5. Histopathologic indexes

The pathological type and grade of the tumor were ascertained via a hollow needle biopsy conducted prior to the initiation of neoadjuvant chemotherapy. Immunohistochemical staining was employed to evaluate the expression of estrogen receptors (ER), progesterone receptors (PR), and HER-2 in breast cancer tissues. ER and PR positivity was defined as the presence of nuclear staining in a minimum of 1% of invasive tumor cells. HER-2 positivity was determined by either an immunohistochemical staining score of 3+ or a score of 2+ in conjunction with positive fluorescence in situ hybridization test results.

### 2.6. Pathological remission criteria

Tumor cell regression within the lesion was evaluated using the Miller–Payne grading system.^[25]^ This system classifies tumor regression into 5 distinct grades based on the reduction in tumor cell density: grade 1 denotes no significant change; grade 2 indicates a high density with <30% reduction; grade 3 corresponds to a 30% to 90% reduction; grade 4 signifies more than a 90% reduction; and grade 5 represents complete tumor disappearance, with no invasive carcinoma detectable microscopically, although ductal carcinoma in situ may still be present.

### 2.7. Peripheral venous blood parameters and nutritional factors

Fasting peripheral venous blood samples were obtained from each patient 1 week prior to and 1 week following the completion of 2 cycles of neoadjuvant chemotherapy (NAC) treatment. Albumin and globulin levels were quantified using a Beckman AU5800 analyzer, and the albumin/globulin (A/G) ratio was subsequently calculated. The body mass index (BMI) was determined based on the patients’ height and weight using the formula BMI = weight ÷ (height)^2^. The ideal body weight was calculated using the formula: Height − 100 − [(Height − 150)/2.5].^[15]^ The percentage change in the A/G ratio after 2 cycles of NAC (ΔA/G2) was calculated as [(A/G before NAC − A/G after 2 cycles of NAC) ÷ A/G before NAC] × 100%. Similarly, the percentage change in BMI (ΔBMI2) was determined as [(BMI before NAC − BMI after 2 cycles) ÷ BMI before NAC] × 100%. The percentage change in the nutritional risk index (NRI) after the second cycle (ΔNRI2) was calculated as [NRI (before NAC) − NRI (after 2 cycles of NAC)] ÷ NRI (before NAC) × 100%. The percentage change in albumin levels (ΔAlbumin2) was calculated as [Albumin (before NAC) − Albumin (after 2 cycles of NAC)] ÷ Albumin (before NAC) × 100%. The percentage change in albumin levels (ΔGlobulin2) was calculated as [Globulin (before NAC) − Globulin (after 2 cycles of NAC)] ÷ Globulin (before NAC) × 100%. Finally, the percentage change in body weight (ΔWeight2) was calculated as [Weight (before NAC) − Weight (after 2 cycles of NAC)] ÷ Weight (before NAC) × 100%.

### 2.8. Statistical analysis

Data analysis was conducted utilizing SPSS Statistics version 27 (Armonk). The normality of measurement data was assessed, and data conforming to a normal distribution were presented as Mean ± standard deviation (*x̄* ± *s*). Group comparisons for normally distributed data were performed using the *t*-test. For data not adhering to a normal distribution, results were expressed as *t M*(*Q*_1_,*Q*_3_), with group comparisons executed via the Mann–Whitney *U* test. Categorical data were presented as frequencies and percentages (n [%]), and group comparisons were made using the chi-square test or Fisher’s exact test, as appropriate. Variables with a *P* value < .2 from the between-group comparisons were included in a multivariate binary logistic regression analysis to identify independent factors influencing pathological complete response (pCR) in breast cancer. Receiver operating characteristic (ROC) curves were also generated. To validate the model’s predictive performance beyond discrimination, we conducted a calibration analysis to evaluate the alignment between predicted probabilities and actual outcomes. Differences with *P* < .05 were considered statistically significant.

## 3. Results

### 3.1. Correlation of clinicopathological parameters with NAC efficacy for breast cancer

A total of 85 patients were enrolled in this study, comprising 39 individuals (45.88%) in the pathological complete response (pCR) cohort and 46 individuals (54.12%) in the non-pathological complete response (non-pCR) cohort. Statistically significant differences were observed between the pCR and non-pCR groups concerning T staging and N staging (*P* = .031, *P* < .001). Patients in the pCR cohort were predominantly classified within the T1, T2, and N1 stages. No statistically significant differences were identified between the 2 groups in terms of age, tumor pathological grade, histological type, or the expression levels of estrogen receptor (ER), progesterone receptor (PR), and HER-2 (*P* > .05), as detailed in Table 1.

**Table 1:** Comparison of pathological parameters between the 2 groups.

Variables		Total	pCR group	Non-pCR group	*t*/*χ*^2^ value	*P* value
Cases		85	39	46	NA	NA
Age (yr), Mean ± SD		56.60 ± 11.13	55.79 ± 10.47	57.28 ± 11.74	*t* = −0.61	.542
Pathological grade (case)	I	21 (24.71)	9 (23.08)	12 (26.09)	χ² = 1.00	.606
	II	50 (58.82)	25 (64.10)	25 (54.35)		
	III	14 (16.47)	5 (12.82)	9 (19.57)		
Histological type (cases)	Invasive ductal carcinoma	47 (55.29)	25 (64.10)	22 (47.83)	–	.274
	Invasive lobular carcinoma	36 (42.35)	13 (33.33)	23 (50.00)		
	Other	2 (2.35)	1 (2.56)	1 (2.17)		
T stage (cases)	T1	32 (37.65)	20 (51.28)	12 (26.09)	–	.031
	T2	46 (54.12)	18 (46.15)	28 (60.87)		
	T3	7 (8.24)	1 (2.56)	6 (13.04)		
N stage (cases)	N1	45 (52.94)	30 (76.92)	15 (32.61)	–	<.001
	N2	35 (41.18)	8 (20.51)	27 (58.70)		
	N3	5 (5.88)	1 (2.56)	4 (8.70)		
ER (cases)	Negative	31 (36.47)	15 (38.46)	16 (34.78)	χ^2^ = 0.12	.725
	Positive	54 (63.53)	24 (61.54)	30 (65.22)		
PR (cases)	Negative	37 (43.53)	18 (46.15)	19 (41.30)	χ^2^ = 0.20	.653
	Positive	48 (56.47)	21 (53.85)	27 (58.70)		
HER-2 (cases)	Negative	36 (42.35)	16 (41.03)	20 (43.48)	χ^2^ = 0.05	.820
	Positive	49 (57.65)	23 (58.97)	26 (56.52)		

– = Fisher exact, χ^2^ = chi-square test, ER = estrogen receptor, HER-2 = human epidermal growth factor receptor-2, NA = not applicable, pCR = pathological complete response, PR = progesterone receptor, SD = standard deviation, *t* = *t*-test.

### 3.2. Correlation between breast mass and the nutrition risk-related index and the efficacy of NAC in breast cancer

Prior to neoadjuvant chemotherapy (NAC), no statistically significant differences were observed between the pathological complete response (pCR) and non-pCR groups with respect to breast tumor long diameter, albumin levels, globulin levels, albumin/globulin (A/G) ratios, weight, body mass index (BMI), and the nutritional risk index (*P* > .05). However, following 2 cycles of NAC, significant differences emerged in albumin levels and the nutritional risk index between the pCR and non-pCR groups (*P* < .05), as illustrated in Table 2. Moreover, the rates of change in breast tumor long diameter (ΔD2), albumin levels (ΔAlbumin), and the nutritional risk index (ΔNRI) after 2 cycles of NAC exhibited statistically significant differences between the pCR and non-pCR groups (*P* < .05), whereas other parameters did not display significant differences (*P* > .05), as detailed in Table 3.

**Table 2:** Comparison of the rate of change in tumor the long diameter of breast mass and NRI between 2 groups of patients before neoadjuvant chemotherapy and after 2 cycles

Variables	Prior to NAC				After 2 cycles NAC			
	pCR group	Non-pCR group	*t*/*Z* value	*P* value	pCR group	Non-pCR group	*t*/*Z* value	*P* value
Cases	39	46	NA	NA	39	46	NA	NA
D (mm), *M (Q*_1_, *Q*_3_)	23.00 (18.00, 32.00)	23.00 (17.25, 31.00)	*Z* = −0.50	.615	12.50 (9.25, 15.00)	15.00 (11.25, 22.75)	*Z* = −1.91	.056
Albumin (g/L), *M (Q*_1_, *Q*_3_)/Mean ± SD	41.20 (38.00, 43.50)	40.35 (37.40, 43.63)	*Z* = −0.90	.368	39.95 ± 3.80	36.55 ± 4.89	*t* = 3.53	<.001
Globulin (g/L), Mean ± SD	28.97 ± 5.36	28.69 ± 3.85	*t* = 0.27	.79	28.58 ± 4.51	27.03 ± 3.42	*t* = 1.76	.083
AGR, *M (Q*_1_, *Q*_3_)/Mean ± SD	1.45 (1.27, 1.56)	1.41 (1.23, 1.55)	*Z* = −0.49	.621	1.43 ± 0.25	1.37 ± 0.23	*t* = 1.17	.247
Weight (kg), Mean ± SD	59.35 ± 7.72	59.63 ± 8.54	*t* = −0.16	.875	58.09 ± 7.46	58.05 ± 8.37	*t* = 0.02	.984
BMI (kg/m^2^), Mean ± SD/*M (Q*_1_, *Q*_3_)	23.34 ± 2.89	24.24 ± 3.10	*t* = −1.38	.172	22.84 ± 2.79	23.60 ± 3.03	*t* = −1.18	.241
NRI, Mean ± SD	106.44 ± 7.33	106.52 ± 7.48	*t* = −0.05	.962	105.51 ± 7.67	97.93 ± 9.86	*t* = 3.90	<.001

AGR = albumin-to-globulin ratio, BMI = body mass index, NA: not applicable, NRI = nutritional risk index, *t* = *t*-test, SD = standard deviation, *Z* = Mann–Whitney test.

**Table 3:** Comparison of the rate of change in tumor the long diameter of breast mass and NRI between 2 groups of patients after 2 cycles of NAC.

Variables	Total	pCR group	Non-pCR group	*t*/χ^2^ value	*P* value
Cases	85	39	46	NA	NA
ΔD2 (%), *M*(*Q*_1_, *Q*_3_)	38.89 (27.78, 52.27)	45.45 (33.74, 62.37)	30.37 (18.48, 42.67)	*Z* = 3.97	<.001
ΔAlbumin2 (%), Mean ± SD	4.90 ± 12.02	1.37 ± 11.95	7.90 ± 11.36	*t* = −2.58	.012
ΔGlobulin2 (%), *M*(*Q*_1_, *Q*_3_)	5.68 (−4.69, 11.34)	4.07 (−3.47, 10.78)	5.80 (−4.73, 11.33)	*Z* = −0.66	.511
ΔAGR 2(%), *M*(*Q*_1_, *Q*_3_)	1.89 (−6.74, 8.01)	1.07 (−7.81, 6.35)	1.92 (−6.38, 10.87)	*Z* = −0.72	.474
ΔWeight2 (%), *M*(*Q*_1_, *Q*_3_)	1.85 (1.67, 2.94)	1.82 (1.57, 2.77)	2.22 (1.68, 3.38)	*Z* = −1.65	.1
ΔBMI2 (%), *M*(*Q*_1_, *Q*_3_)	1.85 (1.67, 2.94)	1.82 (1.57, 2.77)	2.22 (1.68, 3.38)	*Z* = −1.62	.105
ΔNRI2 (%), Mean ± SD	4.62 ± 8.17	0.68 ± 6.62	7.96 ± 6.93	*t* = −4.54	.01

ΔA/G2 = percentage change in A/G ratio after the second NAC cycle, ΔAlbumin2 = percentage change in albumin levels after the second NAC cycle, ΔBMI2 = Percentage change in BMI after the second NAC cycle, ΔD2 = percentage change in tumor maximum diameter after the second NAC cycle, ΔGlobulin2 = percentage change in globulin levels after the second NAC cycle, ΔNRI2 = percentage change in NRI after the second NAC cycle, ΔWeight2 = percentage change in body weight after the second NAC cycle, A/G = albumin/globulin ratio, BMI = body mass index, NA = not applicable, NAC = neoadjuvant chemotherapy, NRI = nutritional risk index, *t* = *t*-test, *Z* = Mann–Whitney test.

### 3.3. Analysis of factors affecting the efficacy of NAC in breast cancer

Initially, a univariate logistic regression analysis was conducted to identify potential predictors of pathological complete response (pCR) in breast cancer patients undergoing neoadjuvant chemotherapy (NAC). Variables such as T stage, N stage, change in tumor maximum diameter (ΔD2), and change in nutritional risk index (ΔNRI2) exhibited statistical significance (*P* < .05) and were selected for further examination. Subsequently, variables with *P* values < .20 from the univariate analysis were included in the multivariate binary logistic regression analysis. This multivariate analysis identified several independent predictors of pCR in breast cancer: T stage (stage II: OR = 2.31, 95% CI: 0.68–7.77, *P* = .177; stage III: OR = 8.22, 95% CI: 0.52–129.58, *P* = .134), N stage (N2: OR = 3.68, 95% CI: 1.08–12.53, *P* = .037), the change rate of the tumor’s longest diameter (ΔD2; OR = 0.95, 95% CI: 0.92–0.99, *P* = .024), and nutritional risk index (ΔNRI2; OR = 1.10, 95% CI: 1.01–1.19, *P* = .032), as detailed in Table 4. The variance inflation factor values for all variables ranged from 1.070 to 1.163, indicating an absence of significant multicollinearity among the predictors.

**Table 4:** Univariate logistics and multivariate logistics regression analysis of factors related to NAC efficacy in breast cancer patients.

Variables	Univariate					Multivariate					VIF
	β	SE	Z	*P*	OR (95%CI)	β	S.E	*Z*	*P*	OR (95%CI)	
T stage											1.081
I					Reference					Reference	
II	0.95	0.47	2.01	.044	2.59 (1.02–6.56)	0.84	0.62	1.35	.177	2.31 (0.68–7.77)	
III	2.30	1.14	2.02	.043	10.00 (1.07–93.44)	2.11	1.41	1.50	.134	8.22 (0.52–129.58)	
N stage											1.085
N1					Reference					Reference	
N2	1.91	0.51	3.73	<.001	6.75 (2.48–18.41)	1.30	0.62	2.09	.037	3.68 (1.08–12.53)	
N3	2.08	1.16	1.79	.074	8.00 (0.82–78.00)	0.55	1.36	0.40	.686	1.73 (0.12–24.77)	
ΔD2 (%)	−0.05	0.01	−3.40	<.001	0.95 (0.92–0.98)	−0.05	0.02	−2.25	.024	0.95 (0.92–0.99)	1.130
ΔBMI2 (%)	0.38	0.20	1.95	.051	1.46 (1.00–2.15)	0.09	0.28	0.33	.745	1.10 (0.63–1.91)	1.070
ΔNRI2 (%)	0.14	0.04	3.71	<.001	1.16 (1.07–1.25)	0.09	0.04	2.15	.032	1.10 (1.01–1.19)	1.163

ΔBMI2 = percentage change in BMI after the second NAC cycle, ΔD2 = percentage change in tumor maximum diameter after the second NAC cycle, ΔNRI2 = percentage change in NRI after the second NAC cycle, ΔWeight2 = percentage change in body weight after the second NAC cycle, BMI = body mass index, CI = confidence interval, NAC = neoadjuvant chemotherapy, NRI = nutritional risk index, OR = odds ratio, SE = standard error, VIF = variance inflation factor.

### 3.4. ROC and calibration curve analysis

**Figure 3.**
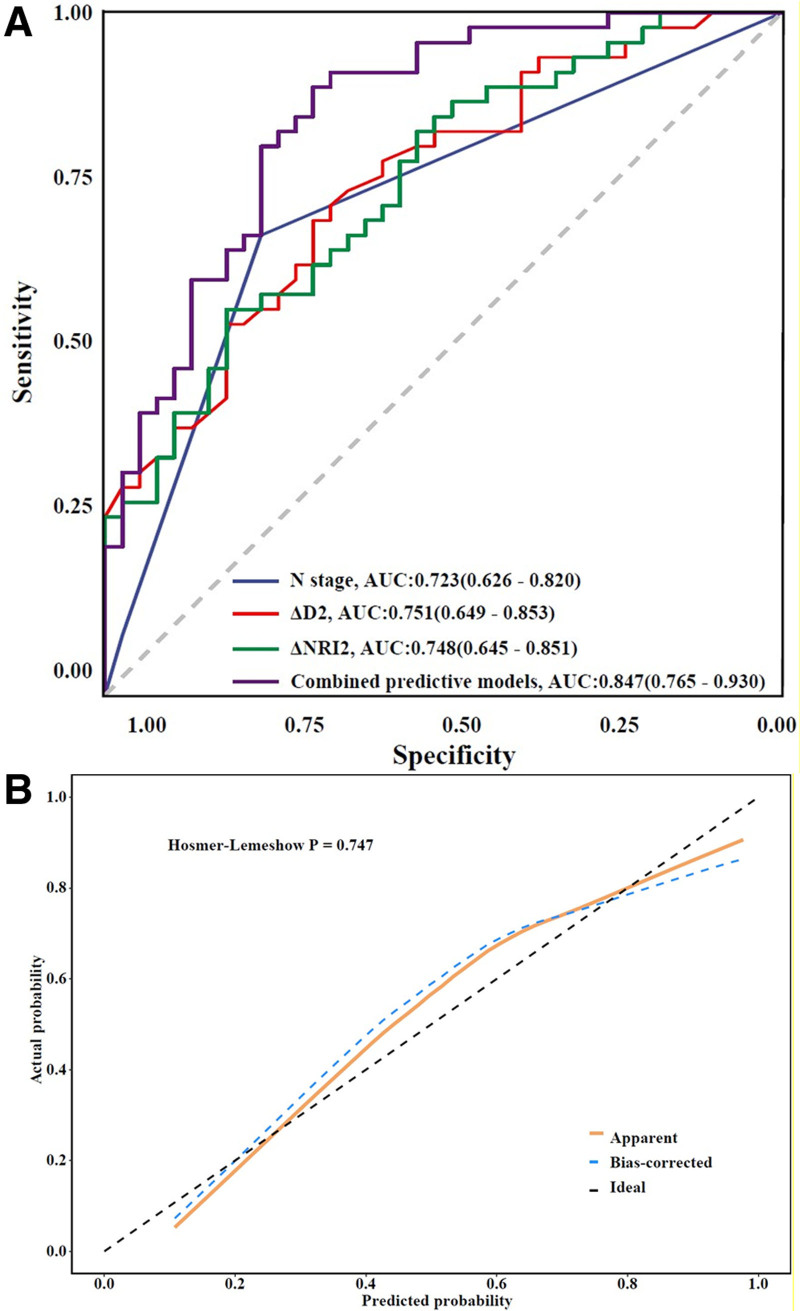
Receiver operating characteristic (ROC) and calibration curve for the multivariate analysis of the pathologic complete response rate after neoadjuvant chemotherapy in breast cancer patients. (A) ROC curve and (B) calibration curve. AUC = area under the curve, ΔD2 = percentage change in tumor maximum diameter after the second NAC cycle; ΔNRI2 = percentage change in NRI after the second NAC cycle. NAC = neoadjuvant chemotherapy, NRI = nutritional risk index, ROC = receiver operating characteristic.

Receiver operating characteristic (ROC) curves were generated for the significant parameters identified in the multivariate analysis. The results revealed that the areas under the ROC curve (AUCs) for N stage, tumor long-axis diameter change rate (ΔD2) and NRI change rate (ΔNRI2) were 0.723, 0.751, 0.748, respectively. The combined diagnostic model yielded an AUC of 0.847 (95% CI: 0.765–0.930), with a sensitivity of 69.2% and a specificity of 89.1%, as depicted in Figure 3A. The calibration curve exhibited a strong concordance between the predicted probabilities and the actual observed outcomes, as illustrated in Figure 3B. Furthermore, the Hosmer–Lemeshow goodness-of-fit test produced a *P* value of .747, suggesting no significant deviation from perfect calibration.

## 4. Discussion

Neoadjuvant chemotherapy (NAC) constitutes a critical therapeutic strategy for the management of locally advanced breast cancer. The primary advantage of NAC lies in its capacity to diminish the size of the primary tumor and metastatic lymph nodes through preoperative systemic intervention. This reduction facilitates a decrease in clinical staging, enhances surgical resection rates, and improves overall prognosis. Nevertheless, the response to neoadjuvant chemotherapy (NAC) among breast cancer patients exhibits significant heterogeneity, with a pathologic complete response (pCR) achieved in only approximately 30% to 40% of cases.^[26]^ Treatment failure is often attributed to either intrinsic or acquired resistance mechanisms. Intrinsic resistance is characterized by preexisting tumor insensitivity prior to the initiation of treatment, which may result from inherent genetic mutations, overexpression of drug efflux transporters such as P-glycoprotein, and factors related to the tumor microenvironment.^[27]^ Conversely, acquired resistance develops during prolonged exposure to treatment and involves adaptive metabolic reprogramming and the selection of resistant cellular clones.^[27]^ Patients who exhibit an inadequate response to NAC are subjected to prolonged exposure to ineffective chemotherapy, which is associated with toxicity, the potential need to convert from breast-conserving surgery to mastectomy, and delays in the initiation of alternative therapies.^[26]^ Therefore, early prediction of NAC efficacy is crucial for timely modification of treatment strategies and optimization of patient outcomes.

According to the Chinese Expert Consensus on Neoadjuvant Treatment of Breast Cancer (2022 Edition), magnetic resonance imaging is recommended as the standard modality for evaluation. However, its application is limited by high costs, complexity, and specific patient contraindications. Computed tomography scanning is unsuitable for repeated monitoring due to the risks associated with radiation exposure.^[28]^ In contrast, ultrasound has emerged as an invaluable assessment tool owing to its noninvasive nature, cost-effectiveness, and high reproducibility. Nevertheless, ultrasound presents limitations in assessing deep or irregular masses and encounters challenges in fully capturing tumor heterogeneity.

Furthermore, while patient nutritional status is an independent factor influencing chemotherapy tolerance and prognosis and partially reflects immune-nutritional status, its predictive capability is limited when considered in isolation. Consequently, this study aims to evaluate the efficacy of neoadjuvant chemotherapy (NAC) at an early stage by employing a predictive model that integrates ultrasound, the nutritional risk index, and other related indicators. This approach seeks to identify patients who are most likely to benefit from NAC, thereby optimizing treatment decisions and improving clinical outcomes.

Dynamic alterations in tumor size serve as critical indicators for evaluating the efficacy of breast cancer treatments. Conventional ultrasound evaluates the effectiveness of neoadjuvant chemotherapy (NAC) by monitoring changes in the target tumor’s size, shape, margins, and internal echoes both pre- and post-treatment. The present study demonstrates that the change in diameter (ΔD2) of breast cancer lesions in the pathological complete response (pCR) group was significantly smaller than that in the non-pCR group. Following 2 cycles of NAC, ΔD2 emerged as an independent predictor of NAC’s therapeutic efficacy in breast cancer. A greater ΔD2 was associated with an increased likelihood of achieving a substantial histological response, as evidenced by a relatively high diagnostic performance (AUC = 0.751). These findings align with those reported by Gao et al,^[29]^ albeit with a slightly reduced diagnostic efficacy, and it is inconsistent with the results of the results presented by Li et al.^[30]^ The primary reason for these discrepancies may be attributed to differences in the study populations. Both this study and that of Gao et al included patients with multiple molecular subtypes, whereas Li et al’s study focused exclusively on triple-negative breast cancer. Furthermore, variations in tumor regression patterns following neoadjuvant chemotherapy (NAC), such as centripetal versus non-centripetal regression, may contribute to measurement discrepancies. In cases of centripetal regression, the measured tumor volume corresponds with the actual reduction in volume. Conversely, in non-centripetal regression, internal tumor cell reduction can cause the measured volume to deviate from the true decrease.

Body mass index (BMI) and serum albumin levels are commonly utilized indicators for assessing nutritional risk.^[31,32]^ Although BMI and serum albumin levels are crucial for evaluating prognosis and treatment outcomes in cancer patients, various non-nutritional factors can influence these measurements. These factors include inflammation, fluid status, renal dysfunction, and hepatic congestion, which may compromise the accuracy of the assessment results.^[33]^ Conversely, the nutritional risk index (NRI), which integrates ideal body weight, serum albumin, and current body weight, provides a more comprehensive evaluation of a patient’s nutritional status. This method helps to mitigate the impact of fluid status, resulting in a more accurate assessment of nutritional risk.^[21]^ In this study, no significant difference was observed in the change in BMI (ΔBMI2) between the 2 groups, whereas NRI (ΔNRI2) were significantly higher in the pathological complete response (pCR) group compared to the non-pCR group. ΔNRI2 was identified as an independent predictor of the efficacy of neoadjuvant chemotherapy (NAC) in breast cancer. Further analysis of its diagnostic performance demonstrated that the change in NRI (ΔNRI, AUC = 0.748) exhibited a relatively high level of diagnostic accuracy. Research by Chen et al also indicated that, in early-stage breast cancer, patients with higher NRI scores had significantly longer average disease-free survival and overall survival (OS) compared to those with lower NRI scores.^[21]^ This research result was consistent with the study of Chen. Further evidence of the clinical value of NRI was demonstrated in breast patient.

The TNM staging system, developed by the American Joint Committee on Cancer, plays a critical role in staging breast cancer and informing treatment decisions. Precise tumor staging is crucial for formulating personalized treatment strategies for patients. This study demonstrates that both the T stage and N stage of breast cancer independently predict pathological complete response (pCR). Earlier N stages are associated with a higher likelihood of achieving pCR, underscoring their significance as indicators for evaluating the effectiveness of neoadjuvant chemotherapy (NAC) in clinical practice. These findings are consistent with those reported by Li et al.^[30]^ Further analysis using the receiver operating characteristic (ROC) curve indicates that N stage exhibit strong diagnostic capabilities, suggesting that clinicians should consider these critical clinicopathological factors when developing treatment plans. This study also developed a predictive model that integrates N stage, ΔD2 and ΔNRI2 to effectively predict the efficacy of NAC for breast cancer, achieving an area under the curve (AUC) of 0.847. These indicators are vital for the diagnosis and assessment of breast cancer patients.

Although albumin levels necessitate intravenous blood sampling, the other indicators are noninvasive tests that can be readily conducted in primary healthcare settings. Consequently, this study underscores the clinical utility and practicality of combining ultrasound with the nutritional risk index for predicting the efficacy of neoadjuvant chemotherapy in breast cancer, advocating for its widespread implementation in healthcare facilities. Despite the important findings of this study, there are limitations. First, the study was a single-center, retrospective analysis with a small sample size, which may lead to model optimism and limit generalizability. As a result, effect estimates and performance metrics might be imprecise. Our findings should be seen as hypothesis-generating and need independent external validation in larger, multicenter cohorts before clinical use. Furthermore, as a retrospective study, it did not comprehensively account for potential confounding variables such as patients’ lifestyles and psychological conditions. Therefore, future research should aim to increase the sample size and employ prospective study designs to enhance the robustness of the findings. Another significant limitation of our study is the absence of comprehensive molecular subtype data for all patients, which prevented the incorporation of molecular subtypes as a predictor variable in our multivariable model.

## 5. Conclusion

Following the second cycle of neoadjuvant chemotherapy (NAC) for breast cancer, variables such as ΔD2, ΔNRI2 and N stage emerge as independent predictors of pathological complete response (pCR), demonstrating substantial diagnostic value. The integration of conventional ultrasound with the nutritional risk index (NRI) as a diagnostic methodology markedly enhances the predictive accuracy regarding the efficacy of NAC in breast cancer. This advancement aids in the effective guidance of clinical treatment and prognosis assessment, potentially reducing the administration of ineffective chemotherapy regimens, fostering the exploration of novel treatment strategies, and ultimately improving overall treatment outcomes for breast cancer. The small sample size and single-center retrospective design limit our findings’ generalizability. Future research should focus on: large-scale, multicenter prospective validation in diverse populations to confirm model robustness; adding biomarkers like circulating tumor cells and ctDNA to improve accuracy; creating dynamic monitoring systems during NAC; and conducting cost-effectiveness analyses for clinical adoption. Rigorous external validation in independent cohorts is crucial before using this model as a standard clinical decision-making tool.

## Acknowledgments

We would like to acknowledge the hard and dedicated work of all the staff that implemented the intervention and evaluation components of the study.

## Author contributions

**Conceptualization:** Yan Shen Liu, Zuo Jie Li.

**Data curation:** Yan Shen Liu, Zuo Jie Li, Hui Wang, Jiao Qun Zhou.

**Formal analysis:** Jiao Qun Zhou.

**Investigation:** Hui Wang, Jiao Qun Zhou.

**Methodology:** Zuo Jie Li, Hui Wang, Jiao Qun Zhou.

**Project administration:** Yan Shen Liu, Jiao Qun Zhou.

**Resources:** Zuo Jie Li, Hui Wang, Jiao Qun Zhou.

**Software:** Zuo Jie Li, Jiao Qun Zhou.

**Supervision:** Hui Wang.

**Validation:** Yan Shen Liu.

**Writing – original draft:** Yan Shen Liu, Zuo Jie Li, Hui Wang, Jiao Qun Zhou.

**Writing – review & editing:** Yan Shen Liu, Zuo Jie Li.
